# Supplementary material for: Complications following surgeries for endometriosis: A systematic review protocol
Source: PLoS One. 2023 May 23;18(5):e0285929. doi: 10.1371/journal.pone.0285929 (PMC10204940; doi:10.1371/journal.pone.0285929)
Supplement: S1 Appendix — (PDF) [file pone.0285929.s002.pdf]

S1 Appendix

Search strategy for Medline (Ovid)

**Concept 1: ENDMETRIOSIS**	
1.	Endometrios* OR Chocolat* cyst$ OR Adenomyoses OR adenomyosis uteri OR endometrial adenoma OR endometriosis interna OR endometriosis, stroma or internal endometriosis OR stroma endometriosis OR stromal endometriosis OR uterine adenomyomatosis OR uterine adenomyosis OR uterus adenomyosis OR endometrios#s OR endometrioma? OR adenomyos#s OR adenomyoma? OR adenometrit#s OR adenomyosit#s OR adenomyometrit#s
2.	Endometriosis"[Mesh] OR "Adenomyosis"[Mesh]
**3.**	**1 OR 2**
**Concept 2: SURGERY**	
4.	Surger* Special*, Colon and Rectal OR Surger*, Colorectal OR Colon and Rectal Surger* Special* OR Colon Surger* Special* OR Special*, Colon Surger* OR Surger* Special*, Colon OR Proctolog* OR Special*, Rectal Surger* OR Surger* Specialty, Rectal OR Rectal Surger* Special* OR coloproctotom* OR proctocolonic surger* OR surger*, colorectal OR Surgery, General OR Surger* OR abdomen surger* OR abdominal operation* OR digestive system surger* OR digestive system surgical procedure* OR surger*, abdominal OR upper abdomen surger* OR abdomen laparotom* OR abdominal laparotom* OR abdomen laparoscop* OR abdominal laparocop* OR Computer-Assisted Surger* OR Surger*, Computer-Assisted OR Surger*, Computer Assisted OR Computer Assisted Surger* OR Computer-Aided Surger* OR Computer Aided Surger* OR Surger*, Computer-Aided OR Surger*, Computer Aided OR Surger*, Image-Guided OR Image-Guided Surger* OR Surger*, Image Guided OR Image Guided Surger* OR Surgical Navigation* OR Navigation*, Surgical OR surger*, elective OR Surgical Procedure*, Elective OR Elective Surgical Procedure OR Procedure*, Elective Surgical OR emergency operation* OR operation*, emergency OR surger* emergency OR surger*, emergency OR surgical emergency OR micro-surger* OR microscale surger* OR microscopic surger* OR microsurgical OR mini-invasive surger* OR mini-invasive surgical procedure* OR minimally invasive surgical method* OR minimally invasive surgical technique* OR surger*, minimally invasive OR Surgical Procedure*, Minimal OR Surgical Procedure*, Minimal Access OR Surgical Procedure*, Minimally Invasive OR Procedure*, Minimally Invasive Surgical OR Minimal Surgical Procedure* OR Minimally Invasive Surger* OR Surger*, Minimally Invasive OR Procedure*, Minimal Surgical OR Procedure*, Minimal Access Surgical OR Minimally Invasive Surgical Procedure* OR Minimal Access Surgical Procedure* OR minor surgical procedure* OR surgery, minor OR minor surger* OR surgical procedure*, minor OR pelvic surger* OR pelvis operation* OR pubis symphysiotom* OR symphysiotomy OR gynaecologic surger* OR gynaecological operation* OR gynaecological surger* OR gynaecology surger* OR gynecologic operation* OR gynecologic surgical procedure* OR gynecological operation* OR gynecological surger* OR gynecology surger* OR operative gynecology OR operative technique* OR surgical procedure*OR endoscopic resection* OR endoscopic surgical procedure* OR resection*, endoscopic OR surger*, endoscopic OR surger*, video assisted OR surgical procedure*, endoscopic OR video assisted surger* OR video-assisted surger* OR laparoscop*, video OR pelvic endoscop* OR peritoneoscop* OR video laparoscop* OR videolaparoscop*OR hand assisted laparoscopic surger* OR hand-assisted laparoscop* OR Exeresis OR excis$ OR radical excision OR genitourinary surger* OR urogenital surger* OR urogenital surgical procedure* OR urogenital tract surger* OR urologic operation* OR urologic surgical procedure* OR urological operation* OR urological surger* OR Procedure*, Urogenital Surgical OR Surgical Procedure*, Urogenital OR Urogenital Surgical Procedure* OR Operative Procedure* OR Procedure*, Operative OR Surgical Procedure*, Operative OR Procedure*, Operative Surgical OR Procedure*, Surgical OR Surgical Procedure* OR Operative Surgical Procedure* OR Surger*, Ghost OR Ghost Surger* OR Laparoscop* OR Celioscop* OR Peritoneoscop* OR Laparoscop* Surgical Procedure* OR Procedure*, Laparoscop*

	Surgical OR Surger*, Laparoscop* OR Laparoscop* Surger* OR Laparoscop* Assisted Surger* OR Surger*, Laparoscop* Assisted OR Surgical Procedure*, Laparoscop* OR Endoscope OR Laparotom* OR Minilaparotom* OR Ablation Technique* OR Technique*, Ablation OR ablation OR Adhesiolysis OR adhesioly$ OR Resect* OR Nodulectomy OR presacral neurectomy OR uterosacral nerve ablation or LUNA OR plasmajet or plasma jet OR laser ablation* OR surger*, laser OR Hysterectom* OR Hysterectomies, Vaginal OR Vaginal Hysterectom* OR Colpohysterectom* OR Trachelectomies OR Vaginal Cervicectom* OR Cervicectom*, Vaginal OR Vaginal Trachelectom* OR Trachelectom*, Vaginal OR Cervicectom* OR hysterocolpectomy OR panhysterectomy OR supervaginal amputation OR total hysterectomy OR uterus amputation OR uterus extirpation OR Intraoperative Monitoring OR Obstetric* Surgical Procedure* OR Procedure*, Obstetric* Surgical OR Surgical Procedure*, Obstetric* OR Surger*, Obstetric* OR Obstetric* Surger*
5.	"Colorectal Surgery"[Mesh] OR "Specialties, Surgical"[Mesh] OR "General Surgery"[Mesh] OR "Surgical Procedures, Operative"[Mesh] OR "Laparoscopy"[Mesh] OR "Laparoscopes"[Mesh] OR "Laparoscopes"[Mesh] OR "Endoscopes"[Mesh] OR "Laparotomy"[Mesh] OR "Ablation Techniques"[Mesh] OR "Ambulatory Surgical Procedures"[Mesh] OR "Elective Surgical Procedures"[Mesh] OR "Minimally Invasive Surgical Procedures"[Mesh] OR "Minor Surgical Procedures"[Mesh] OR "Monitoring, Intraoperative"[Mesh] OR "Obstetric Surgical Procedures"[Mesh] OR "Cystostomy"[Mesh]OR "Pelvic Exenteration"[Mesh] OR "Surgery, Computer-Assisted"[Mesh] OR "Urogenital Surgical Procedures"[Mesh] OR General Surgery/ OR "Margins of Excision"/ or Lymph Node Excision/ OR "Hysterectomy"[Mesh]
**6.**	**4 OR 5**
**Concept 3: COMPLICATION**	
7.	hemorrhage* OR haemorrhage OR blood loss OR bleeding OR Conversion to an Open Surgical Procedure* OR Conversion to an Open Procedure* OR Conversion to an Open sergur* OR Mortalit*, death OR Organ injur* OR reentry surgery OR re-entry surgery OR second look laparoscopy OR second look operation OR second-look surgery OR surgical follow*up OR postoperative reintervention OR Second Look Surger* OR Surger*, Second-Look OR Surger*, Second Look OR patient readmission OR readmission* OR readmission rate OR rehospitalization OR Readmission, Patient OR 30-Day Readmission* OR 30 Day Readmission OR Thirty Day Readmission* OR Readmission, Thirty Day OR Hospital Readmission* OR Readmission*, Hospital OR Stay Length* OR Hospital Stay* OR stay*, Hospital OR LOL OR Complication*, Peroperative OR Peroperative Complication* OR Complication*, Intraoperative OR Intraoperative Complication* OR Injur*, Surgical OR Surgical Injur* OR Complication* OR complication* rate* OR outcome* OR Complication*, Postoperative OR Postoperative Complication*
8.	"Hemorrhage"[Mesh] OR "Conversion to Open Surgery"[Mesh] OR "Mortality"[Mesh] OR "Recurrence"[Mesh] OR "Second-Look Surgery"[Mesh OR "Patient Readmission"[Mesh] OR "Length of Stay"[Mesh] OR "Intraoperative Complications"[Mesh] OR "Postoperative Complications"[Mesh]
**9.**	**7 OR 8**
10.	exp animals/ not humans.sh.
**11.**	**3 AND 6 AND 9 NOT 10**
