# Supplementary material for: Complications following surgeries for endometriosis: A systematic review protocol
Source: PLoS One. 2023 May 23;18(5):e0285929. doi: 10.1371/journal.pone.0285929 (PMC10204940; doi:10.1371/journal.pone.0285929)
Supplement: S2 Appendix — (PDF) [file pone.0285929.s003.pdf]

S2 Appendix

Data extraction form

**Review information**	
Reviewer identification	
Date of review	
Contact with the author necessary	□ Yes<br>□ No<br>Date communication sent:
Response	□ None<br>□ Yes, but no data<br>□ Yes, with additional data received
Notes	

**Study identification**	
Study ID	
Title	
Lead author and contact detail	
Source	□ Journal article<br>□ Abstract<br>□ Conference proceeding<br>□ Other (e.g., unpublished data)<br>Specify:
Journal	
Year of publication	
Volume	
Page (start-end)	
Language	□ English<br>□ French<br>□ Other<br>Specify:
Notes	

**Eligibility of study in the review**	
Inclusion criteria	
Study design (prospective and retrospective cohort studies or trials)	□ Yes □ No
Intervention (endometriosis surgery)	□ Yes □ No
Outcome (perioperative and/or postoperative complications)	□ Yes □ No

Study period	◻ Yes ◻ No
Start date of the study	
End date of the study	
Exclusion criteria	
Gynecological cancer diagnosis	◻ Yes ◻ No
Concomitant benign gynecologic surgeries	◻ Yes ◻ No
Eligibility confirmed	◻ Yes ◻ No

**Study characteristics**	
Aim of the study	
Country in which the study conducted	◻ United States<br>◻ UK<br>◻ Canada<br>◻ France<br>◻ Not specify<br>◻ Other<br>Specify:
Funding Source	◻ Governmental<br>◻ Industry<br>◻ Hospital<br>◻ None<br>◻ Other
Study design	◻ Randomized controlled trial<br>◻ Non-randomized experimental study<br>◻ Retrospective Cohort study<br>◻ Prospective Cohort study<br>◻ N/A
Type of study	◻ Single center<br>◻ Cohort
Method of recruitment of participants	◻ Phone<br>◻ Mail<br>◻ Voluntary<br>◻ Clinic patients<br>◻ Questionnaire<br>◻ Not specified<br>◻ Other<br>Specify:
Setting (type of center)	◻ LEVEL I<br>◻ LEVEL II<br>◻ LEVEL III<br>◻ LEVEL IV<br>◻ Not Specified<br>◻ Other<br>Specify:

<table>
<tr><td>Ethical approval</td><td colspan="4">□ Yes
□ No
□ Not specify</td></tr>
<tr><td>Informed consent obtained?</td><td colspan="4">□ Yes
□ No
□ Not specify</td></tr>
<tr><td>Method selection</td><td colspan="4">□ Consecutive
□ Random
□ Voluntary
□ Not specified
□ Other</td></tr>
<tr><td>Participants Inclusion criteria (list them)</td><td colspan="4"></td></tr>
<tr><td>Exclusion criteria (list them)</td><td colspan="4"></td></tr>
<tr><td>Comparator</td><td colspan="4">□ Placebo
□ Standard of care
□ Other intervention
□ None
□ Other</td></tr>
<tr><td rowspan="3">Surgical intervention</td><td>□ MIH</td><td colspan="2">□ LSH
□ TLH
□ LAVH
□ VH
□ RATH</td><td>□ With BSO
□ Without BSO</td></tr>
<tr><td>□ TAH</td><td colspan="2"></td><td>□ With BSO
□ Without BSO</td></tr>
<tr><td>□ Major conservative surgery</td><td>□ laparoscopy
□ laparotomy</td><td>□ shaving
□ discoid excision
□ colorectal segmental resection
□ resection of the bladder
□ segmental ureteral resection
□ cystotomy
□ cystectomy
□ lesion ablation (plasma energy or laser)
□ electrocoagulation
□ combined technique (excision and ablation)</td><td>□ With BSO
□ Without BSO</td></tr>
</table>

			□ pelvic denervation<br>□ endometrioma drainage<br>□ lesion excision	
	□ Minor conservative surgery	□laparoscopy<br>□laparotomy	□ biopsy<br>□ minor adhesiolysis	
Concomitant complex surgeries	□ Hysterectomy<br>□ Colorectal segmental resection<br>□ Bladder resection<br>□ Segmental ureteral resection<br>□ None			
Study's primary outcome				
Study's secondary outcome(s)				
Follow-up duration after the surgery				
Possible conflicts of interest for study authors				

*MIH: minimally invasive hysterectomy; TAH: total abdominal hysterectomy; Laparoscopic supracervical hysterectomy (LSH); Total laparoscopic hysterectomy (TLH); Laparoscopically assisted vaginal hysterectomy (LAVH); Vaginal hysterectomy (VH); Robotic-assisted total hysterectomy; Bilateral salpingo-oophorectomy (BSO)

**Patients Flow**	
Screened (n)	
Eligible (n)	
Excluded (n)	
Randomized (if applicable)	
Lost to follow-up (n)	
Exclusion from analysis justified?	

**Patient characteristics**	
Age (mean; SD; range)	
BMI (mean; SD)	
Smoker, n (if the author specified)	
Comorbidity	n:<br>□ not available
List the comorbidities	

Stages of endometriosis	□ stage I (minimal)<br>□ stage II (mild)<br>□ stage III (moderate)<br>□ stage IV (severe)<br>□ endometrioma<br>□ not available
Indication of surgery	□ infertility<br>□ pain<br>□ not available
The author specified the history of surgery	□ yes<br>□ no
Participants with surgical history, n	
Participants without surgical history, n	
Gave birth prior to the surgery, n	
Nulliparity n (%)	
Race/ethnicity (list according to study)	

* N/A : not available

**Outcome**	**Major conservative surgery (with BSO)**	**Major conservative surgery (without BSO)**	**Minor conservative surgery**	**MIH (With BSO)**	**MIH (Without BSO)**	**TAH (with BSO)**	**TAH (without BSO)**	**Concomitant surgeries**
**Composite outcome**								
**Intraoperative complication**								
**Post-operative complications (overall)**								
**Post-operative complications in first month**								
**Post-operative complications in three months**								
**Grade I-II CD (minor complication) ***								
**Grade III- V DC (major complications) ***								
**Length of surgery**								
**Readmission**								
**Length of hospitalization**								
**Open surgery conversion**								
**Post-operative reintervention**<br>Laparoscopic revision<br>Laparotomy								
**Infection**<br>Wound infection<br>Urinary tract infection<br>Vaginal infection<br>Abscess								

Pyelonephritis<br>Cellulitis<br>Sepsis<br>Fever/pyrexia								
**Vascular repair**								
**Venous thromboembolism**<br>Thrombophlebitis<br>Pulmonary embolus								
**Pneumothorax**								
**Damage to blood vessels**								
**Genitourinary tract injury**<br>Cystotomy<br>Ureteral injury (transection)<br>Vesicovaginal fistula<br>Ureterovaginal fistula<br>Urinary retention<br>Bladder atony<br>Voiding dysfunction<br>Self-catheterization (< and > 30 days)								
**Gastrointestinal tract injury**<br>Injury to gastrointestinal tract<br>Anastomotic dehiscence/anastomotic leak syndrome<br>Rectovaginal fistula<br>Rectal stenosis<br>Rectal perforation<br>Ileostomy								
**Vaginal cuff dehiscence**								
**Bleeding**<br>Blood transfusion								
Hematoma<br>Vascular injury								
**Nerve injury**								
**Repair to bladder**								
**Repair to ureter**								
**Repair to bowel**								
**Other complications¶**								

* Grade I: was assigned to any deviation from the normal postoperative course without the need for pharmacological treatment or surgical, endoscopic, and radiological interventions. Allowed therapeutic regimens are drugs as antiemetics, antipyretics, analgetics, diuretics and electrolytes, and physiotherapy. This grade also includes wound infections opened at the bedside; Grade II: Requiring pharmacological treatment with drugs other than such allowed for Grade I complications. Blood transfusions and total parenteral nutrition are also included; Grade III: requiring surgical, endoscopic, or radiological intervention; Grade III-a: intervention not under general anesthesia; Grade III-b: intervention under general anesthesia; Grade IV: life-threatening complication (including CNS complications) requiring IC/ICU-management; Grade IV-a: single organ dysfunction Grade IV-b: multi organ dysfunction; Grade V: death of a patient.

¶ Other complications included accidental cut, puncture, perforation, or hemorrhage during medical care and surgical procedures as the cause of abnormal reaction of patient or later complication

Quality assessment

Descriptive analyses			
**Bias domain**	**Signaling questions**	**Response**	**note**
Bias in selection of participants into the study	1. Was selection of participants into the study (or into the analysis) based on participant characteristics observed after the start of intervention (surgery)?<br>**If N/PN to 2.1:** go to 2.4	□Y<br>□PY<br>□N<br>□PN<br>□Unclear	
	2. **If Y/PY to 1**: Were the post-intervention variables that influenced selection likely to be associated with intervention?	□Y<br>□PY<br>□N<br>□PN<br>□Unclear	
	3 **If Y/PY to 2**: Were the post-intervention variables that influenced selection likely to be influenced by the outcome or a cause of the outcome?	□Y<br>□PY<br>□N<br>□PN<br>□Unclear	
	4. Do follow-up time coincide for most participants?	□Y<br>□PY<br>□N<br>□PN<br>□Unclear	
	5. **If Y/PY to 2 and 3, or N/PN to 4**: Were analysing techniques used likely to correct for the presence of selection biases?	□Y<br>□PY<br>□N<br>□PN<br>□Unclear	
	**Risk of bias judgement**	**□Low risk of bias**<br>**□High risk of bias**<br>**□Unclear**	
Bias due to missing data	1 Were outcome data available for all, or nearly all, participants?	□Y<br>□PY<br>□N<br>□PN<br>□Unclear	
	2 Were participants excluded due to missing data on intervention status?	□Y<br>□PY<br>□N<br>□PN<br>□Unclear	
	3 Were participants excluded due to missing data on other variables needed for the analysis?	□Y<br>□PY<br>□N<br>□PN<br>□Unclear	
	4 **If PN/N to 1, or Y/PY to 2 or 3**: Are the proportion of participants and reasons for missing data provided and acceptable?	□Y<br>□PY<br>□N<br>□PN<br>□Unclear	

	5 **If PN/N to 1, or Y/PY to 2 or 3**: Is there evidence that results were robust to the presence of missing data?	□Y<br>□PY<br>□N<br>□PN<br>□Unclear	
	**Risk of bias judgement**	**□Low risk of bias**<br>**□High risk of bias**<br>**□Unclear**	
Bias in classification of interventions	1 Was the intervention clearly defined?	□Y<br>□PY<br>□N<br>□PN<br>□Unclear	
	2 Was the information used to define the intervention recorded at the start of the intervention?	□Y<br>□PY<br>□N<br>□PN<br>□Unclear	
	3 In case there were multiple types of surgeries studied, would classification of intervention have been affected by knowledge of the outcome or risk of the outcome?	□Y<br>□PY<br>□N<br>□PN<br>□Unclear	
	**Risk of bias judgement**	**□Low risk of bias**<br>**□High risk of bias**<br>**□Unclear**	
Bias due to deviations from intended interventions	1. Were there deviations from the intended intervention beyond what would be expected in usual practice?	□Y<br>□PY<br>□N<br>□PN<br>□Unclear	
	2. **If Y/PY to 1**: Were these deviations from intended intervention likely to have affected the outcome?	□Y<br>□PY<br>□N<br>□PN<br>□Unclear	
	3. Were important co-interventions used?	□Y<br>□PY<br>□N<br>□PN<br>□Unclear	
	4. Were changes in the intervention documented?	□Y<br>□PY<br>□N<br>□PN<br>□Unclear	
	5. Would changes in the intervention be likely to introduce bias?	□Y<br>□PY<br>□N<br>□PN<br>□Unclear	

	**Risk of bias judgement**	**□Low risk of bias**<br>**□High risk of bias**<br>**□Unclear**	
Bias in measurement of outcomes	1 Could the outcome measure have been influenced by knowledge of the intervention received?	□Y<br>□PY<br>□N<br>□PN<br>□Unclear	
	2 Were outcome assessors aware of the intervention received by study participants?	□Y<br>□PY<br>□N<br>□PN<br>□Unclear	
	3 Were the methods of outcome assessment comparable across intervention groups?	□Y<br>□PY<br>□N<br>□PN<br>□Unclear	
	4 Were any systematic errors in measurement of the outcome related to intervention received?	□Y<br>□PY<br>□N<br>□PN<br>□Unclear	
	**Risk of bias judgement**	**□Low risk of bias**<br>**□High risk of bias**<br>**□Unclear**	
Bias in selection of the reported result	1. ... multiple outcome *measurements* within the outcome domain?	□Y<br>□PY<br>□N<br>□PN<br>□Unclear	
	2 ... multiple *analyses* of the intervention-outcome relationship?	□Y<br>□PY<br>□N<br>□PN<br>□Unclear	
	3 ... different *subgroups*?	□Y<br>□PY<br>□N<br>□PN<br>□Unclear	
	**Risk of bias judgement**	**□Low risk of bias**<br>**□High risk of bias**<br>**□Unclear**	
**Overall Risk of bias**		**□Low risk of bias**<br>**□High risk of bias**<br>**□Unclear**	

Potential determinants analyses			
**Bias domain**	**Signaling questions**	**Response**	**note**
Bias in classification of determinants	1 Were the determinants considered clearly defined?	□Y<br>□PY<br>□N<br>□PN<br>□Unclear	
	2 Was the information used to define the determinants considered recorded prior to the surgery?	□Y<br>□PY<br>□N<br>□PN<br>□Unclear	
	3 Would classification of the determinants considered have been affected by knowledge of the outcome or risk of the outcome?	□Y<br>□PY<br>□N<br>□PN<br>□Unclear	
	4 Were the measurements of determinants prone to misclassification?	□Y<br>□PY<br>□N<br>□PN<br>□Unclear	
	**Risk of bias judgement**	**□Low risk of bias<br>□High risk of bias<br>□Unclear**	
Bias due to confounding of the association between determinants and complications	1 Is there potential for confounding of the association between determinants and complications of surgeries in this study?<br>**If N/PN to 1:** the study can be considered to be at low risk of bias due to confounding and no further signalling questions need be considered	□Y<br>□PY<br>□N<br>□PN<br>□Unclear	
	**Questions relating to baseline confounding only**		
	1.4. Did the authors use an appropriate analysis method that controlled for all the important confounding domains?	□Y<br>□PY<br>□N<br>□PN<br>□Unclear	
	1.5. **If Y/PY to 1.4**: Were confounding domains that were controlled for measured validly and reliably by the variables available in this study?	□Y<br>□PY<br>□N<br>□PN<br>□Unclear	
	1.6. Did the authors control for any post-intervention variables that could have been affected by the intervention?	□Y<br>□PY<br>□N<br>□PN<br>□Unclear	
	**Risk of bias judgement**	**□Low risk of bias<br>□High risk of bias<br>□Unclear**	
